# Precision cancer therapy is impacted by oncogene-dependent epigenome remodeling

**DOI:** 10.1038/s41698-017-0005-2

**Published:** 2017-03-20

**Authors:** Feng Liu, Paul S. Mischel, Webster K. Cavenee

**Affiliations:** 1 National Research Center for Translational Medicine (Shanghai), State Key Laboratory of Medical Genomics, Rui-Jin Hospital, Shanghai Jiao Tong University School of Medicine, Shanghai, 200025 China; 2 Ludwig Institute for Cancer Research, La Jolla, CA 92093 USA; 3 Department of Pathology, UCSD School of Medicine, La Jolla, CA 92093 USA; 4 Moores Cancer Center, UCSD School of Medicine, La Jolla, CA 92093 USA; 5 Department of Medicine, UCSD School of Medicine, La Jolla, CA 92093 USA

## Abstract

The cancer genome provides the blueprint for identifying oncogenic mutations driving tumor growth and these mutant proteins and pathways are the targets for precision cancer therapies. However, many oncogenes are capable of reprogramming the landscape of active portion of the genome, commonly known as the epigenome. This creates fluidity, and thereby heterogeneity, that demands consideration of this additional layer of complexity for effective therapeutic design and application. Molecular dissection of the epigenome may identify oncogene-induced, actionable vulnerabilities, broadening the spectrum of precision oncology treatments.

## Introduction

When the War on Cancer was announced in 1971, neoplastic disease was largely an unknown and ill-described enemy. Today, almost every common cancer has been portrayed in exquisite detail using whole-genome sequencing and various genomic analyses, providing an increasingly clear picture of their genetic aberrations.^1^ Genomic characterization of the cancer genome is especially empowering for cancer researchers and clinicians, since the molecular mechanisms underlying the initiation and maintenance of tumorigenesis are critically dependent on the function of oncogenes (mutant, active cancer-promoting genes) and tumor suppressors (mutant, loss of function cancer-inhibiting genes).^2^ Genetic manipulation of one or a few oncogenes in animal models is often sufficient to cause tumor relapse in vivo, implying that drugs targeting specific driver mutations will have greater specificity and less toxicity than more conventional cytotoxic therapies. This idea of precision oncology gained international attention through the development and clinical success of the tyrosine kinase inhibitor Gleevec (imatinib, STI-571) in treatment of chronic myelogenous leukemia (CML).^3^ The availability of large genomic data sets has also allowed investigation of the effects of cancer genotypes on drug sensitivity and resistance.^4^ Thus, the knowledge of cancer genomes has been well positioned to allow for comprehensive characterization of driver mutations in a given tumor, and serve the foundation to rationally design drug combinations to target each driver mutation in patients.

With a complete catalogue of oncogenic mutations in sight, the current stage of cancer research echoes the time when the draft of the human genome revealed, for the first time, an exhaustive list of all the coding sequences (i.e., genes) in the genome.^5, 6^ Nevertheless, an informative lesson learned from the Human Genome Project (HGP) is that just knowing the sequence of the genome does not immediately lead to a mechanistic understanding of the molecular program deployed by the genome to guide the development of body form and function. Recognizing this barrier between genotype and phenotype, the HGP was followed by consortium studies to map the epigenome—a term used to describe the landscape of chromatin regions containing genes and gene regulatory elements in different cell types and developmental stages.^7^ These epigenomic studies have unraveled a much richer view of how different parts of the genome coordinate to control cell type specification and differentiation.^8^ Similarly, studies are under way to functionally annotate the cancer epigenome.^9^ In this perspective, we highlight recent advances in this area. We further argue that certain epigenetic changes in cancers are mechanistically linked to the activity of oncogenic mutations, and that understanding the downstream consequences of this may provide previously unsuspected and valuable targets for therapy.

## The landscape of the epigenome

**Fig. 1:**
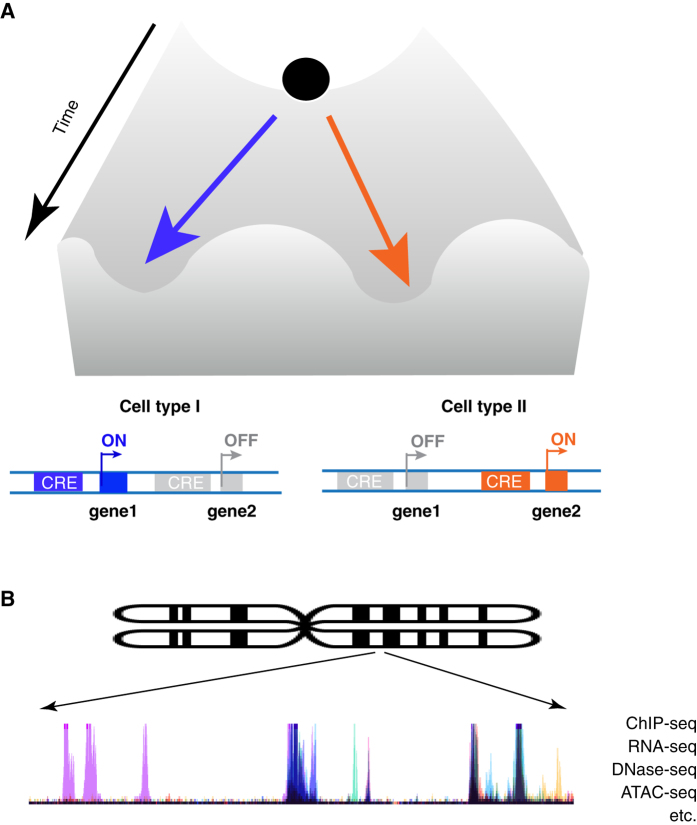
**a** Schematic illustration adapted from C.H. Waddington’s "epigenetic landscape": as development proceeds, a stem/progenitor falls down a likelihood hill toward terminal differentiation at the bottom. At the molecular level, cell fate specification and differentiation are controlled by select expression of genes in the genome. **b** The pattern of biochemical activities on the chromosome can be characterized by next-generation sequencing-based technologies. These patterns provide a comprehensive view of the epigenome

The term of epigenome derives from the metaphor of the epigenetic landscape used by Conrad H. Waddington in the 1940s to explain the developmental pathways that a stem/progenitor cell might take toward differentiation^10^ (Fig. 1a). Later studies showed that such cell fate plasticity is due to the selected expression of a small portion of the genes from the genome in different cellular contexts, which allows for a single genome to potentially guide the appearance of different specialized cell types in a multicellular organism.^11^

Starting from the early 1960s, studies in both prokaryotes and eukaryotes showed that gene regulation is fundamentally carried out by two types of intracellular factors.^12^ The first are the transcription factors (TFs), which are DNA-binding proteins localized in the nucleus. The second are the *cis*-regulatory elements (CREs), which are non-coding sequences in chromatin that selectively recruit TFs through short (6–20 bps) nucleotide sequence motifs. In the eukaryotic genome, there are three major classes of CREs. The first two, called promoters and enhancers, cooperatively recruit a diverse array of TFs to activate gene expression, whereas the third class, called insulators, act as gene expression repressors by interfering with the interaction between promoters and enhancers.^13^

The human genome encompasses 1391 TFs^14^ and approximately a million CREs.^8^ In principle, the different combination of these TFs and CREs active in a cell can account for the many and varied possible cell types and subtypes during development.^15^ Over the past decade, next-generation sequencing (or high-throughput sequencing)-based techniques have enabled the determination of the global patterns of TF binding and chemical modifications of the chromatin (e.g., DNA methylation and histone acetylation/methylation) and that these differ in each cell at different stages and tissues. These patterns thus represent the epigenomic landscape, a panoramic view of the active portion of the genome^16^ (Fig. 1b).

## Oncogenes and the cancer epigenome are intertwined

**Fig. 2:**
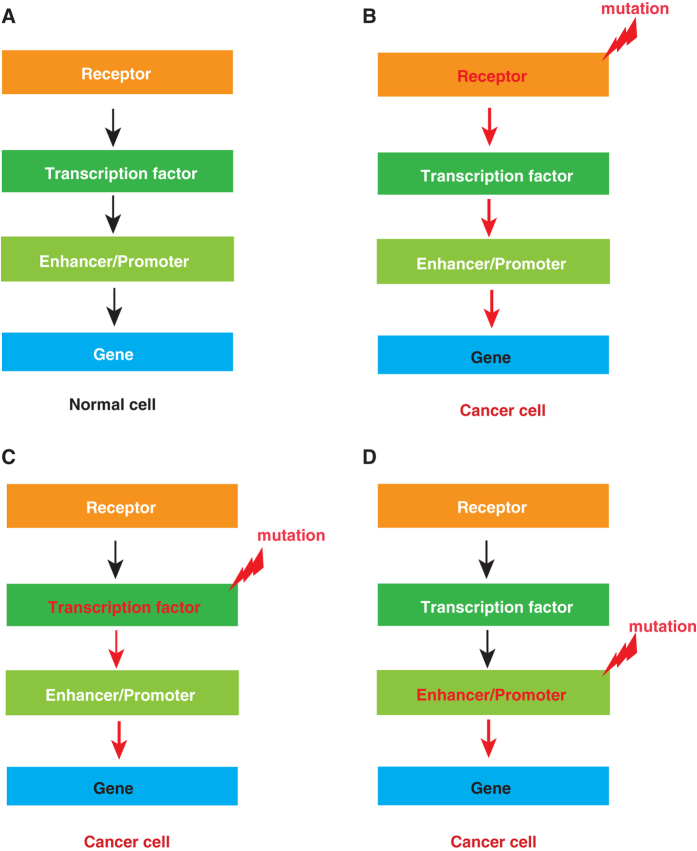
**a** The hierarchy of signal-regulated expression of genes. Signal receptors activate distinct transcription factors through chemical modification of the latter (e.g., phosphorylation, acetylation, methylation, ubiquitination etc.). TFs bind with DNA sequence motifs in enhancers and promoters, which are associated with specific genes in the genome. **b–d** Oncogenic mutations are common among signal receptors and signal-responsive TFs, enhancers, and promoters. When the factors at the higher level of the regulatory hierarchy are mutated, those at lower levels are often mobilized to activate aberrant gene expression programs in cancer cells

Like essentially all active molecules in the cell, TFs, and CREs are both subject to the action of other regulatory molecules (Fig. 2a). In particular, the gene regulatory function of some TFs is contingent upon the activity of certain signaling receptors on the cell surface. Signaling receptor proteins are transmembrane proteins, whose extracellular domain can bind with ligands secreted from adjacent cells. The ligand-receptor binding can then trigger the intracellular domain of the receptor protein to either directly or indirectly activate of the TFs in the nucleus. TFs, in turn, cooperate with enhancers bearing their binding sites to stimulate the transcription of downstream target genes. Through this chain of interactions, these enhancers and promoters serve as signal-transducers to regulate signal-dependent gene expression, which controls context-dependent cell fate specification and differentiation in early development and maintains tissue homeostasis in adults.^15^

In cancer, it has been noted that signaling receptors and signal-transducing TFs are frequent targets of oncogenic lesions. For example, receptors for all major signaling pathways (RTK, Notch, TGF-beta, nuclear receptors etc.) are recurrent driver mutations.^17^ As receptors normally sit at the top of the hierarchy of gene regulatory networks (Fig. 2a), it is perhaps not surprising that oncogenic mutant receptors are generally gain of function mutations, which contribute to tumorigenesis by globally changing the gene expression profiles in the cell^18^ (Fig. 2b). Mutant TFs are also common cancer drivers, which differ from their wild-type counterparts by activating different sets of genes or activating the same genes in different tissue types or at different developmental stages^19^ (Fig. 2c). Finally, mutations in enhancers, promoters, and insulators disrupt the specificity of gene expression in different cell types, leading to aberrant gene regulatory activities in cancer (Fig. 2d).^20, 21^

Furthermore, because the cascade of signal-regulated gene expression programs relies on intercalated molecular interactions, when higher-ranking molecules in the regulatory hierarchy are mutated, no mutations are absolutely required in those at lower ranks to participate in the tumorigenic gene expression program (Figs. 2b–d). In other words, when an upstream transcriptional regulator goes awry, many seemingly normal downstream regulators are involuntarily recruited to contribute to aberrant gene expression programs in cancer. For example, in a recent study, a recurrent driver mutation named EGFRvIII—which encodes a truncated, constitutively active epidermal growth factor receptor (EGFR)—was shown to activate and repress thousands of enhancer-associated genes involved in glioblastoma development.^22^ This EGFRvIII-dependent reprogramming of the epigenetic landscape is, inlarge part, due to the ability of this oncogene to activate the expression of two TFs, SOX9 and FOXG1. Similar roles of oncogenes in remodeling the cancer epigenome has also been studied in other cancers such as chronic lymphocytic leukemia^23^ and Ewing sarcoma.^24^

## The signatures of the cancer epigenome

Key to the recent explosive growth in the field of genomics is the development of high-throughput sequencing-based methods that allow global identification of patterns that correlate with transcriptional activities. These methods include those mapping the binding of TFs (TF ChIP-seq), methylation of DNA (bisulfite-seq), covalent modification of histones (histone mark ChIP-seq), and accessible regions of chromatin (DNase-seq and ATAC-seq).^16^ The high-throughput capacity of these methods have enabled systematical characterization of major CREs—i.e., enhancers, promoters, and insulators—in the genome, because the function and location of these elements are positively correlated with these features of the epigenome.

Given the specific functional linkage between enhancers, promoters and genes, genome-wide characterization of CREs has recently emerged as an entry point to investigate gene regulatory programs specific to different cancer cell types. For example, the finding of focal amplification of two enhancers differentially active in lung adenocarcinoma and endometrial carcinoma provided the telltales of distinct regulatory mechanism of *c-MYC* overexpression in these two types of cancers.^25^ Moreover, from a global map of active enhancers and promoters in a cell, the upstream *trans*-regulators can then be deduced from the TF-binding motifs enriched in these CREs. In this way, the epigenetic signature of CREs can be exploited to decode the gene regulatory mechanisms in cancer. Recently, this strategy has served as the basis for the study of transcriptional programs underlying the tumorigenecity of glioblastoma,^22, 26^ as well as in the study of the subgroup-specific cellular origins of medulloblastoma.^27^

## Targeting the cancer epigenome

The fundamental requirement for developing targeted cancer therapy is that the drug target is both necessary and sufficient to drive cancer cell proliferation and survival. However, many cancers appear to have an uncanny ability to escape the reliance on individual oncogenes. In some cases, this is because pre-existing and acquired mutations change the drug target site.^28^ In others, drug resistance is due to many levels of redundancy among oncogenes and/or feedback loops that compensate for the loss of the product of a particular oncogene, which generally fall in the category of reprogramming of the molecular interaction networks in the cancer cell.^29^

To overcome the resistance against drugs targeting the oncogenic pathways in the cell membrane and cytoplasm, an alternative is to target gene regulatory molecules in the nucleus. Oncogenic TFs were among the earliest cancer “drivers” cloned in the 1980s. Yet TF proteins are notoriously “undruggable” by small molecules, largely due to the relatively large size of the domains of DNA binding and transcription activation in each TF. At present, only a small number of approaches are underway to identify new compounds to target TFs whose activities require them to directly interact with other proteins.^30, 31^ On the other hand, compounds have been recently developed that target epigenetic chromatin structure modulators. For example, two histone deacetylase inhibitors—vorinostat (suberoylanilide hydroxamic acid) and romidepsin (depsipeptide)—were approved in 2010 by the US FDA for the treatment of relapsed cutaneous T-cell lymphoma.^32^ At about the same time, two small molecules, JQ1 and I-BET, were found to selectively inhibit the histone-binding activity of members of the bromodomain and extra-terminal (BET) family (BRD2, BRD3, BRD4 and BRDT), which are critical components of a transcription cofactor complex that binds to acetylated lysines of histones at enhancer loci.^33, 34^ Both JQ1 and I-BET are potent repressors of transcription and both exhibit strong anti-tumor activities in cancers that appear to be “addicted” to elevated transcriptional activities, including AML,^35, 36^ T-ALL,^37^ mixed lineage leukemia, diffusive large B cell lymphoma,^38^ glioblastoma,^22^ medulloblastoma,^39^ and KRAS-mutant non-small-cell lung cancer.^40^ Another compound named THZ1, a covalent inhibitor of cyclin-dependent kinase CDK7, was found to effectively suppresses gene promoters and exhibited potent anti-cancer effect in T-ALL,^41^ MYC-N-amplified neuroblastoma,^42^ small-cell-lung cancer,^43^ and triple-negative breast cancer.^44^ Finally, GSKJ4, an ethyl ester derivative of the H3K27 demethylase inhibitor GSKJ1, decreases histone H3K27 demethylase JMJD3 and thus increases cellular H3K27 methylation.^45^ This reversion of K27 methylation level appeared to alleviate the change of transcriptional activities in K27M mutant tumors, and in doing so abolishes the tumorigenic capacity of in H3K27M pediatric brainstem glioma cells and T-ALL.^45, 46^ These novel anti-cancer drugs targeting chromatin modulators thus mark the dawn of a new era of “epigenetic therapy”.^47, 48^

## Future outlook

The success of targeted cancer therapy in CML and the available cancer genome databases introduced a tremendous amount of optimism for the development of precision cancer therapies. In conjunction with recent efforts to delineate the epigenetic landscape of the cancer genome,^8, 16^ the impact of mutations on the function of the cancer genome can be studied in more depth. It is anticipated that, in the following decade, not only the potency but also the specificity of these new generation anti-cancer drugs will be scrutinized in detail. With further refinement, these drugs may finally bring the study of genetic and epigenetic mechanisms of gene regulation into fruition in the clinic.

Looking ahead, three pressing challenges need to be confronted before realizing the potential of cancer genome studies. First, tumors are generally composed of mixed populations of cells, as a result of elevated mutation rates in cancer cells (because of aberrant DNA damage repair mechanisms), tumor compositions that include both cancer cells and neighboring normal cells, and the asynchronous (de)differentiation of tumor cells. To distinguish specific drug targets in each population, it is necessary to extend cancer genomic analyses to the level of studying single cancer cells in each tumor.^49, 50^ Second, due to the lack of patient samples for experimentation, comprehensive epigenomic profiling of cancers has not been widely used to study cancers thus far. In the future, new protocols that require only small numbers of cells need to be developed to simultaneously study the cancer genome and epigenome. Speed and cost-effectiveness of these methods will also be critical for their application in clinical settings to aid cancer diagnoses and treatment efficacy evaluations. Finally, further studies are required to assess the specificity and efficacy of the drugs targeting the epigenome. As precision oncology approaches become a standard part of the care of cancer patients, one can envision a future in which DNA sequencing, transcriptional and epigenetic profiling are used to develop precise therapies, including combinations, most likely to suppress disease based on that individual’s tumor composition.

## References

[1] Garraway LA, Lander ES (2013). Lessons from the cancer genome. Cell.

[2] Hanahan D, Weinberg RA (2011). Hallmarks of cancer: the next generation. Cell.

[3] Druker BJ (2008). Translation of the Philadelphia chromosome into therapy for CML. Blood.

[4] Iorio, F., Knijnenburg, T. A., Vis, D. J., Bignell, G. R., Menden, M. P. & Schubert, M. *et al.* A landscape of pharmacogenomic interactions in cancer. *Cell* **166**, 740–754 (2016). 10.1016/j.cell.2016.06.017 PMC4967469 27397505

[5] Venter JC, Adams MD, Myers EW, Li PW, Mural RJ, Sutton GG (2001). The sequence of the human genome. Science.

[6] Lander ES, Linton LM, Birren B, Nusbaum C, Zody MC, Baldwin J (2001). Initial sequencing and analysis of the human genome. Nature.

[7] Bernstein BE, Meissner A, Lander ES (2007). The mammalian epigenome. Cell.

[8] Consortium EP (2012). An integrated encyclopedia of DNA elements in the human genome. Nature.

[9] Beck S, Bernstein BE, Campbell RM, Costello JF, Dhanak D, Ecker JR (2012). A blueprint for an international cancer epigenome consortium. a report from the aacr cancer epigenome task force. Cancer Res..

[10] Waddington CH (2012). The epigenotype. 1942. Int. J. Epidemiol..

[11] Goldberg AD, Allis CD, Bernstein E (2007). Epigenetics: a landscape takes shape. Cell.

[12] Jacob F, Monod J (1961). Genetic regulatory mechanisms in the synthesis of proteins. J. Mol. Biol..

[13] Levine M, Cattoglio C, Tjian R (2014). Looping back to leap forward: transcription enters a new era. Cell.

[14] Vaquerizas JM, Kummerfeld SK, Teichmann SA, Luscombe NM (2009). A census of human transcription factors: function, expression and evolution. Nat. Rev. Genet..

[15] Peter I. S., Davidson E. H.*Genomic Control Process : Development and Evolution*. 448 (Academic, 2015)

[16] Rivera CM, Ren B (2013). Mapping human epigenomes. Cell.

[17] Tamborero D, Gonzalez-Perez A, Perez-Llamas C, Deu-Pons J, Kandoth C, Reimand J (2013). Comprehensive identification of mutational cancer driver genes across 12 tumor types. Sci. Rep.

[18] Weinstein JN, Collisson EA, Mills GB, Shaw KR, Ozenberger BA, Cancer Genome Atlas Research N (2013). The cancer genome atlas pan-cancer analysis project. Nat. Genet..

[19] Bhagwat AS, Vakoc CR (2015). Targeting transcription factors in cancer. Trends in Cancer.

[20] Sur, I. & Taipale, J. The role of enhancers in cancer. *Nat. Rev. Cancer* **16**, 483–493 (2016). 10.1038/nrc.2016.62 27364481

[21] Flavahan WA, Drier Y, Liau BB, Gillespie SM, Venteicher AS, Stemmer-Rachamimov AO (2016). Insulator dysfunction and oncogene activation in IDH mutant gliomas. Nature.

[22] Liu F, Hon GC, Villa GR, Turner KM, Ikegami S, Yang H (2015). EGFR mutation promotes glioblastoma through epigenome and transcription factor network remodeling. Mol. Cell.

[23] Rendeiro AF, Schmidl C, Strefford JC, Walewska R, Davis Z, Farlik M (2016). Chromatin accessibility maps of chronic lymphocytic leukaemia identify subtype-specific epigenome signatures and transcription regulatory networks. Nat. Commun.

[24] Tomazou EM, Sheffield NC, Schmidl C, Schuster M, Schonegger A, Datlinger P (2015). Epigenome mapping reveals distinct modes of gene regulation and widespread enhancer reprogramming by the oncogenic fusion protein EWS-FLI1. Cell Rep.

[25] Zhang X, Choi PS, Francis JM, Imielinski M, Watanabe H, Cherniack AD (2016). Identification of focally amplified lineage-specific super-enhancers in human epithelial cancers. Nat. Genet..

[26] Suva ML, Rheinbay E, Gillespie SM, Patel AP, Wakimoto H, Rabkin SD (2014). Reconstructing and reprogramming the tumor-propagating potential of glioblastoma stem-like cells. Cell.

[27] Lin CY, Erkek S, Tong Y, Yin L, Federation AJ, Zapatka M (2016). Active medulloblastoma enhancers reveal subgroup-specific cellular origins. Nature..

[28] McGranahan N, Swanton C (2015). Biological and therapeutic impact of intratumor heterogeneity in cancer evolution. Cancer Cell.

[29] Furnari FB, Cloughesy TF, Cavenee WK, Mischel PS (2015). Heterogeneity of epidermal growth factor receptor signalling networks in glioblastoma. Nat. Rev. Cancer.

[30] Hart J. R., Garner A. L., Yu J., Ito Y., Sun M., Ueno L. *et al.* Inhibitor of MYC identified in a Krohnke pyridine library. *Proceedings of the National Academy of Sciences of the United States of America*. **111**, 12556–12561 (2014). 10.1073/pnas.1319488111 PMC4151726 25114221

[31] Hagenbuchner J, Ausserlechner MJ (2016). Targeting transcription factors by small compounds-Current strategies and future implications. Biochem. Pharmacol..

[32] Lemoine M, Younes A (2010). Histone deacetylase inhibitors in the treatment of lymphoma. Discov. Med..

[33] Filippakopoulos P, Qi J, Picaud S, Shen Y, Smith WB, Fedorov O (2010). Selective inhibition of BET bromodomains. Nature.

[34] Nicodeme E, Jeffrey KL, Schaefer U, Beinke S, Dewell S, Chung CW (2010). Suppression of inflammation by a synthetic histone mimic. Nature.

[35] Dawson MA, Gudgin EJ, Horton SJ, Giotopoulos G, Meduri E, Robson S (2014). Recurrent mutations, including NPM1c, activate a BRD4-dependent core transcriptional program in acute myeloid leukemia. Leukemia.

[36] Zuber J, Shi J, Wang E, Rappaport AR, Herrmann H, Sison EA (2011). RNAi screen identifies Brd4 as a therapeutic target in acute myeloid leukaemia. Nature.

[37] Roderick JE, Tesell J, Shultz LD, Brehm MA, Greiner DL, Harris MH (2014). c-Myc inhibition prevents leukemia initiation in mice and impairs the growth of relapsed and induction failure pediatric T-ALL cells. Blood.

[38] Chapuy B, McKeown MR, Lin CY, Monti S, Roemer MG, Qi J (2013). Discovery and characterization of super-enhancer-associated dependencies in diffuse large B cell lymphoma. Cancer Cell.

[39] Bandopadhayay P, Bergthold G, Nguyen B, Schubert S, Gholamin S, Tang Y (2014). BET bromodomain inhibition of MYC-amplified medulloblastoma. Clin. Cancer Res.

[40] Shimamura T, Chen Z, Soucheray M, Carretero J, Kikuchi E, Tchaicha JH (2013). Efficacy of BET bromodomain inhibition in kras-mutant non-small cell lung cancer. Clin. Cancer Res.

[41] Kwiatkowski N, Zhang T, Rahl PB, Abraham BJ, Reddy J, Ficarro SB (2014). Targeting transcription regulation in cancer with a covalent CDK7 inhibitor. Nature.

[42] Chipumuro E, Marco E, Christensen CL, Kwiatkowski N, Zhang T, Hatheway CM (2014). CDK7 inhibition suppresses super-enhancer-linked oncogenic transcription in MYCN-driven cancer. Cell.

[43] Christensen CL, Kwiatkowski N, Abraham BJ, Carretero J, Al-Shahrour F, Zhang T (2014). Targeting transcriptional addictions in small cell lung cancer with a covalent CDK7 inhibitor. Cancer Cell.

[44] Wang Y, Zhang T, Kwiatkowski N, Abraham BJ, Lee TI, Xie S (2015). CDK7-dependent transcriptional addiction in triple-negative breast cancer. Cell.

[45] Ntziachristos P, Tsirigos A, Welstead GG, Trimarchi T, Bakogianni S, Xu L (2014). Contrasting roles of histone 3 lysine 27 demethylases in acute lymphoblastic leukaemia. Nature.

[46] Hashizume R, Andor N, Ihara Y, Lerner R, Gan H, Chen X (2014). Pharmacologic inhibition of histone demethylation as a therapy for pediatric brainstem glioma. Nat. Med..

[47] Egger G, Liang G, Aparicio A, Jones. PA (2004). Epigenetics in human disease and prospects for epigenetic therapy. Nature.

[48] Helin K, Dhanak D (2013). Chromatin proteins and modifications as drug targets. Nature.

[49] Patel AP, Tirosh I, Trombetta JJ, Shalek AK, Gillespie SM, Wakimoto H (2014). Single-cell RNA-seq highlights intratumoral heterogeneity in primary glioblastoma. Science.

[50] Venteicher AS, Tirosh I, Hebert C, Escalante L, Martuza RL, Nahed BV (2016). 142 Genetic and nongenetic determinants of cellular architecture in IDH1-mutant oligodendrogliomas and astrocytomas using single-cell transcriptome analysis. Neurosurgery.

